# BRCC36 Deubiquitinates HMGCR to Regulate the Interplay Between Ferroptosis and Pyroptosis

**DOI:** 10.1002/advs.202304263

**Published:** 2024-01-04

**Authors:** Haiyan Wang, Long Shu, Cairui Lv, Na Liu, Yao Long, Xintong Peng, Huli Ling, Tania Tao, Jun Tang, Yan Cheng, Shuang Liu, Desheng Xiao, Yongguang Tao

**Affiliations:** ^1^ Key Laboratory of Carcinogenesis and Cancer Invasion (Central South University Ministry of Education) Department of Pathology Xiangya Hospital Central South University Hunan 410078 China; ^2^ Academy of Biomedical Engineering Kunming Medical University Kunming 650500 China; ^3^ NHC Key Laboratory of Carcinogenesis Cancer Research Institute and School of Basic Medicine Central South University Changsha Hunan 410078 China; ^4^ Hunan Key Laboratory of Early Diagnosis and Precision Therapy Department of Thoracic Surgery Second Xiangya Hospital Central South University Changsha 410011 China; ^5^ Department of Pharmacy The Second Xiangya Hospital Central South University Changsha 410011 China; ^6^ Department of Oncology Institute of Medical Sciences National Clinical Research Center for Geriatric Disorders Xiangya Hospital Central South University Changsha Hunan 410008 China; ^7^ Department of Pathology Xiangya Hospital Central South University Changsha Hunan 410008 China; ^8^ Hunan Key Laboratory of Cancer Metabolism Hunan Cancer Hospital and The Affiliated Cancer Hospital of Xiangya School of Medicine Central South University Changsha Hunan 410031 China

**Keywords:** BRCC36, endoplasmic reticulum, ferroptosis, HMGCR, mitochondria, pyroptosis

## Abstract

Various forms of programmed cell death (PCD) exhibit distinct characteristics depending on their specific molecular mechanisms, and there are interactions among these different forms. Ferroptosis, which is related to autophagy and apoptosis, has an unknown potential interaction with pyroptosis. This study revealed a mutually antagonistic relationship between ferroptosis and pyroptosis, with 3‐hydroxy‐3‐methylglutaryl‐coenzyme A reductase (HMGCR) playing a key role in their interaction. It is found that HMGCR predominantly localized to mitochondria during ferroptosis but shifted to the endoplasmic reticulum following treatment with a pyroptosis inducer. Furthermore, this study demonstrated that BRCC36 (BRCA1/BRCA2‐containing complex subunit 36) deubiquitinated HMGCR in a manner dependent on deubiquitinating enzyme (DUB) activity, and inhibited ferroptosis and promoted pyroptosis. Moreover, as an oncogene in hepatocellular carcinoma (HCC), BRCC36 promoted cancer cell proliferation, migration, invasion, and tumor growth. Thiolutin, an inhibitor of BRCC36, effectively suppressed the interaction between BRCC36 and HMGCR, leading to the inhibition of HCC growth. Therefore, targeting BRCC36 can offer a novel and promising therapeutic strategy for HCC treatment. In conclusion, these findings provide new theoretical evidence for further characterizing tumor heterogeneity and offer new molecular targets for the diagnosis and treatment of HCC.

## Graphical abstract

The crosstalk between different types of cell death remains poorly known. This study identifies a mutually antagonistic relationship between ferroptosis and pyroptosis, with HMGCR playing a key role in their interaction. Moreover, BRCC36 inhibits ferroptosis and promotes pyroptosis by deubiquitinating HMGCR to promote tumor growth, which can provide a novel target for HCC treatment.

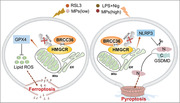

## 1 Introduction

Cell death is of great significance in regulating several physiological processes and maintaining body homeostasis. Recent studies have revealed the existence of crosstalk among different forms of programmed cell death (PCD).^[^ ^1^ ^]^ Ferroptosis is an iron‐dependent cell death triggered by the substantial accumulation of oxidized phospholipids in cellular membranes, which is distinct from other types of PCD in terms of cell morphology, biochemical characteristics, and genetic regulation.^[^ ^2^ ^]^ Various studies have demonstrated that ferroptosis is associated with physiological and pathological processes, including neuronal degeneration and various types of cancer such as liver cancer, lung cancer, colon cancer, and pancreatic cancer. In multiple cancer types, ferroptosis is hindered through the activation of systemic Xc transporters, increased metabolism of glutathione (GSH), enhanced activity of glutathione peroxidase (GPX4), and inhibition of lipid peroxidation and iron metabolism.^[^ ^3^, ^4^ ^]^ Accumulation of lipid peroxides is a key hallmark of ferroptosis, and GPX4 serves as the fundamental regulatory molecule that facilitates the conversion of GSH into oxidized glutathione (GSSG), thereby eliminating harmful lipid peroxides within the cell.^[^ ^5^ ^]^ HMGCR, which is a rate‐limiting enzyme of mevalonate production, inhibits ferroptosis by enhancing the synthesis of GPX4 and coenzyme Q10 (CoQ_10_). According to the latest studies, the GSH‐GPX4, GCH1 (GTP‐dependent cyclohydrolase 1)‐BH4 (tetrahydrobiopterin), NADPH‐FSP1 (ferroptosis suppressor protein 1)‐CoQ_10_, and DHODH (dihydroorotate dehydrogenase)‐CoQ_10_ pathways are major antioxidant systems that regulate cellular susceptibility to ferroptosis. Cells undergo ferroptosis upon inhibition of GPX4, FSP1, GCH1, and other antioxidant systems.^[^ ^6^, ^7^, ^8^, ^9^, ^10^, ^11^, ^12^ ^]^ Emerging studies have demonstrated the crosstalk between ferroptosis and various forms of programmed cell death. Certain selective types of autophagy, such as ferritinophagy, lipophagy, clockophagy, mitophagy, and chaperone‐mediated autophagy, have been found to promote ferroptosis by increasing the levels of free iron or lipid accumulation.^[^ ^4^, ^13^, ^14^, ^15^, ^16^ ^]^ Our previous study reported that a nuclear long non‐coding RNA LINC00618 promotes ferroptosis in an apoptosis‐dependent manner.^[^ ^17^ ^]^ Additionally, the ferroptosis‐inducing agent artesunate induces endoplasmic reticulum (ER) stress response, which plays an important role in mediating the crosstalk between ferroptosis and apoptosis.^[^ ^18^ ^]^ Moreover, there is a significant interplay between necroptosis and ferroptosis, where the activation of necroptosis can contribute to the induction of ferroptosis in ischemic stroke.^[^ ^19^ ^]^

Pyroptosis is a lytic form of programmed cell death driven by gasdermin family proteins, which results in cytoplasmic swelling and the release of inflammatory mediators, such as IL‐1β and IL‐18.^[^ ^20^, ^21^, ^22^ ^]^ Other than DFNB59, gasdermins (GSDMA‐GSDME) act as the primary executioners of pyroptosis. Proteolytic cleavage of gasdermins releases N‐terminal fragments that form large oligomeric pores in the membrane, which further disrupt cell membrane integrity, ultimately causing cell death.^[^ ^23^, ^24^, ^25^, ^26^ ^]^ GSDMD can be cleaved by inflammatory caspases when cells are subjected to external stimuli including bacteria, viruses, toxins, and generate an active N‐terminal fragment (GSDMD‐N) in the cytosol. The GSDMD‐N recognizes and inserts into the cell membrane, contributing to pore formation and pyroptotic cell death.^[^ ^23^, ^24^ ^]^ It It has been shown that granzyme can also induce pyroptosis by directly cleaving gasdermins. Pyroptosis is not only associated with viral diseases but also has a significant impact on cancers and central nervous system disorders.^[^ ^27^ ^]^ Emerging research indicates that the role of pyroptosis in cancer is multifaceted. On the one hand, pyroptosis has the potential to impede tumor development. Pyroptosis, on the other hand, can stimulate the establishment of a cancer‐friendly microenvironment that promotes tumor growth.^[^ ^28^, ^29^, ^30^ ^]^ Previous studies have shown that 4‐HNE (4‐hydroxynonenal), the main product of lipid peroxidation, has been found to suppress NLRP3 (NOD‐like receptor thermal protein domain associated protein 3) inflammasome activation and pyroptosis in macrophages.^[^ ^31^ ^]^ Iron‐induced reactive oxygen species (ROS) can oxidize the mitochondrial outer membrane protein Tom20, which promotes the recruitment of Bax to the mitochondria and stimulates caspase‐3/GSDME‐mediated pyroptosis.^[^ ^32^ ^]^ In summary, there is a potential link between ferroptosis and pyroptosis, but the underlying interaction and mechanism remain obscure. The deubiquitination enzyme BRCC36, encoded by the BRCC3 gene, is a member of the JAMM family. It specifically enables the removal of the K63 polyubiquitin chain (K63‐UB) that is linked to the target protein. However, it remains to be explored whether BRCC36 has additional functions beyond DNA damage repair and immune response.^[^ ^33^ ^]^

In the present study, we explored the relationship between ferroptosis and pyroptosis and found that they had an antagonistic interaction. Notably, we found that HMGCR played a crucial role in the interaction between ferroptosis and pyroptosis. We also demonstrated that BRCC36 deubiquitinated HMGCR in a manner dependent on the activity of DUB, and inhibited ferroptosis while promoting pyroptosis. Moreover, thiolutin, an inhibitor of BRCC36, slowed the progression of HCC, suggesting that BRCC36 could be as a therapeutic target for HCC. Collectively, this study provides new theoretical evidence for further characterizing tumor heterogeneity and offers new molecular targets for the diagnosis and treatment of HCC.

## 2 Results

### 2.1 HMGCR is a Key Regulator of the Interaction Between Ferroptosis and Pyroptosis

**Figure 1:**
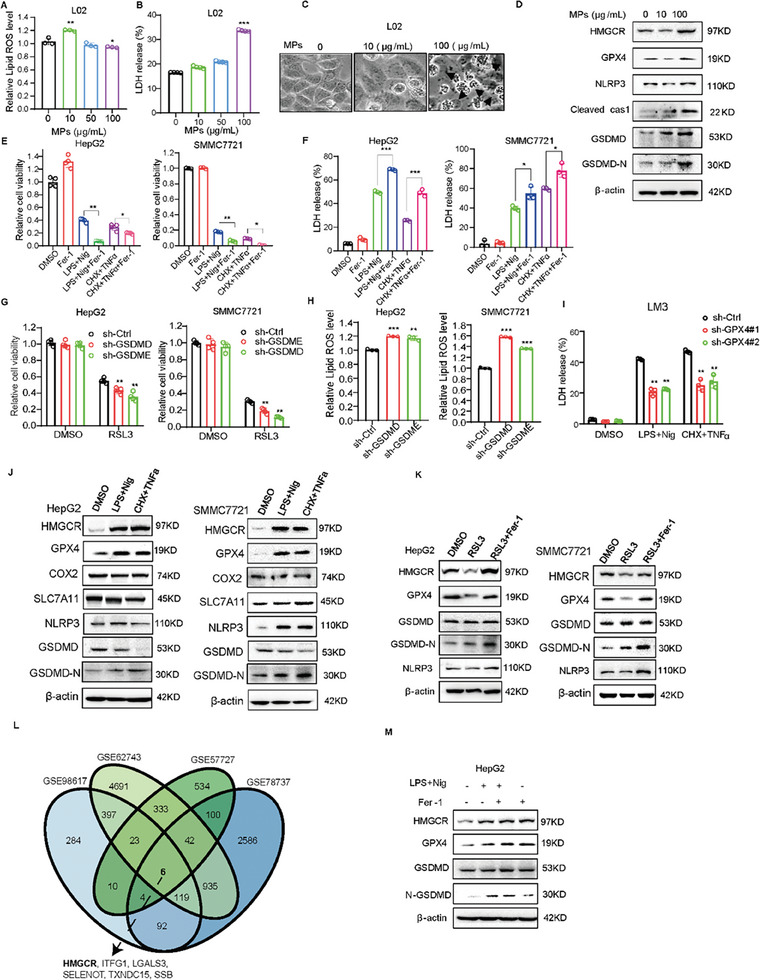
HMGCR is a key regulator of the correlation between ferroptosis and pyroptosis. The relative level of lipid ROS was measured by Flow cytometry A), and the release of LDH was measured by using LDH kits B), and the pyroptotic morphology was observed C) in L02 cells treated with the various concentrations of MPs. Black arrows represent pyroptotic cells. D) The expression levels of proteins associated with ferroptosis and pyroptosis in L‐02 cells treated with different concentrations of MPs. Analysis of cell viability E) and the LDH release F) in HepG2 and SMMC7721 cells treated with pyroptosis inducers, including LPS and Nig, CHX and TNFα, and ferrostatin‐1. Analysis of cell viability G) and relative lipid ROS levels H) in HepG2 and SMMC7721 cells with knockdown of GSDMD/GSDME and RSL3 treatment. I) The release of LDH was measured by using LDH kits in LM3 cells with GPX4 knockdown treated with pyroptosis inducers, including LPS and Nig, CHX and TNFα. J) The expression levels of proteins associated with ferroptosis and pyroptosis by using western blot assay in HepG2 and SMMC7721 cells treated with pyroptosis inducers LPS and Nig, CHX and TNFα. K) The expression levels of proteins associated with ferroptosis and pyroptosis in HepG2 and SMMC7721 cells treated with RSL3 and Fer‐1. L) Venn diagram showed the overlapping number of genes that are simultaneously correlated with both ferroptosis and pyroptosis scores, as determined by the ssGSEA algorithm, in the GSE98617, GSE62743, GSE57727, and GSE78737 datasets. M) The expression levels of proteins associated with ferroptosis and pyroptosis in HepG2 cells treated with the pyroptosis inducer LPS and Nig and ferroptosis inhibitor Fer‐1. Data are presented as mean ± S.D (*n* = 3), and the P value was calculated using two‐sided unpaired Student's t‐tests (A, B, E, to I). *P < 0.05, **P < 0.01, ***P < 0.001.

A previous study has demonstrated that polystyrene microplastics (MPs) have regulatory effects on ferroptosis and pyroptosis, which subsequently contribute to liver damage. However, the underlying molecular mechanisms remain to be fully investigated.^[^ ^34^ ^]^ To assess the correlation between ferroptosis and pyroptosis, we first treated hepatocytes with different concentrations of MPs. We found that the low concentration of MPs increased the level of intracellular lipid ROS (a surrogate of ferroptosis) (**Figure** **1**A), whereas the high concentration of MPs triggered the release of LDH (Figure 1B) and induced cell death with typical pyroptotic morphology, characterized by cell swelling and plasma membrane blebbing (Figure 1C). We utilized western blot assay to analyze the expression of ferroptosis markers (e.g., GPX4, HMGCR) and pyroptosis regulators (e.g., GSDMD, NLRP3, cleaved caspase1), and found the expression of NLRP3, cleaved caspase1, N‐GSDMD were increased after treatment with high concentrations of MPs. However, the expression of HMGCR and GPX4 decreased at low concentrations (Figure 1D). We also treated HepG2 cells with various concentrations of lipopolysaccharide (LPS), and found that LPS could induce ferroptosis and pyroptosis under different concentrations (Figure S1A–C, Supporting Information), indicating that ferroptosis and pyroptosis could be triggered by the same inducers, they do not occur simultaneously. This demonstrates that ferroptosis and pyroptosis might be mutually exclusive. Next, we assessed the association between the key genes of ferroptosis (SLC7A11, GPX4) and pyroptosis (CASP1, GSDMD) in the TCGA‐LIHC database. We found a strong positive correlation between the expression of GPX4 and GSDMD, indicating that the interaction between ferroptosis and pyroptosis is mutually exclusive (Figure S1D, Supporting Information).

We further verified the crosstalk between ferroptosis and pyroptosis. We detected the expression of GSDMD in several HCC cancer cells and found that GSDMD was expressed (Figure S1E, Supporting Information). We treated liver cancer cells with the pyroptosis inducers LPS and nigericin (Nig), CHX and TNFα,^[^ ^35^ ^]^ and found that these inducers markedly induced cell death, exhibiting typical pyroptotic morphology (Figure S1F, Supporting Information). We then used pyroptosis inducers and the ferroptosis inhibitor Fer‐1(ferrostatin‐1) or Lip‐1 (liproxstatin‐1) to treat liver cancer cells, and found that Fer‐1 or Lip‐1 exacerbated cell death and LDH release triggered by pyroptosis inducers (Figure 1E,F; Figure S1G,H, Supporting Information). Importantly, the knockdown of core pyroptosis regulators GSDMD and GSDME significantly accelerated RSL3‐induced cell death (Figure 1G; Figure S1I, Supporting Information) and lipid ROS levels (Figure 1H) in HepG2 and SMMC7721 cells. Moreover, the knockdown of GSDMD or GSDME also increased cell death triggered by the ferroptosis inducer ML162 and inhibited LDH release caused by pyroptosis inducers (Figure S1J–L, Supporting Information). We further utilized pyroptosis inducers (LPS and Nig, CHX and TNFα) to treat LM3 cells with stable GPX4 knockdown, and found that knockdown of GPX4 inhibited LDH release induced by inducers (Figure 1I; Figure S1M, Supporting Information). Moreover, during the treatment with pyroptosis inducers, the levels of the ferroptosis‐associated proteins HMGCR and GPX4 were upregulated, while the levels of COX2 and SLC7A11 remained unchanged (Figure 1J). The expression of HMGCR and GPX4 decreased during RSL3 treatment, but the expression of GSDMD‐N, cleaved cspase1, and NLRP3 was elevated following RSL3 and Fer‐1 treatment (Figure 1K). We examined the expression of ferroptosis and pyroptosis‐associated proteins in HepG2 cancer cells after treatment with pyroptosis inducers and the apoptosis trigger vincristine (VCR), and found that the expression of HMGCR, GPX4, cleaved caspase1 and GSDMD‐N was significantly upregulated after treatment with pyroptosis inducers, but not after treatment with the apoptosis trigger VCR (Figure S1N, Supporting Information). These findings suggest that the interaction between ferroptosis and pyroptosis is mutually antagonistic.

Next, we explored the key factors in the crosstalk between ferroptosis and pyroptosis. We first utilized single sample gene set enrichment analysis (ssGSEA) to construct the ferroptosis and pyroptosis scores in GEO datasets (GSE98617, GSE62743, GSE57727, and GSE78737), and evaluated the correlation between the ssGSEA scores and all genes (Figure S10, Supporting Information). Finally, we identified six genes that simultaneously correlated with ferroptosis and pyroptosis scores in four GEO datasets, including HMGCR, ITFG1 (integrin alpha FG‐GAP repeat containing 1), LGALS3 (galectin 3), SELENOT (selenoprotein T), TXNDC15 (thioredoxin domain containing 15), and SSB (small RNA binding exonuclease protection factors la) (Figure 1L). HMGCR could interfere with GPX4 biosynthesis and increase CoQ10 production to inhibit ferroptosis, but whether HMGCR regulates the interplay between ferroptosis and pyroptosis is obscure. As shown in Figure 1J,K, we observed that HMGCR exhibited the most significant alteration in HepG2 and SMMC7721 cells following treatment with pyroptosis or ferroptosis inducers. We found that the level of HMGCR expression was significantly upregulated in a dose‐dependent manner in HepG2 treated with other pyroptosis‐related drugs doxorubicin and talabostat (Figure S1P, Supporting Information). Moreover, we found that the expression of HMGCR was higher in the group of pyroptosis inducer (LPS and Nig) and the ferroptosis inhibitor Fer‐1 (Figure 1M), indicating HMGCR may play a key role in the correlation between ferroptosis and pyroptosis. These results demonstrate that the correlation between ferroptosis and pyroptosis is independent, and HMGCR plays a crucial role in the interaction between ferroptosis and pyroptosis.

### 2.2 HMGCR Promotes Pyroptosis in the Endoplasmic Reticulum and Inhibits Ferroptosis in the Mitochondria

**Figure 2:**
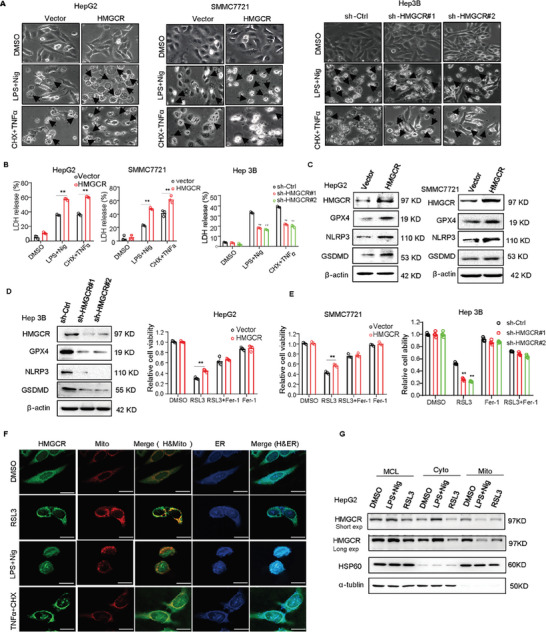
HMGCR promotes pyroptosis in the endoplasmic reticulum and inhibits ferroptosis in the mitochondria. The overexpression or knockdown of liver cancer cells was treated with pyroptosis inducers LPS and Nig, CHX and TNFα. The pyroptotic morphology was observed A). Black arrows represent pyroptotic cells. The release of LDH was measured by using LDH detection kits B). C,D) Western blot assay was performed to detect the expression levels of proteins associated with ferroptosis and pyroptosis in liver cancer cells with HMGCR overexpression or knockdown. E) Cell viability was measured by using the CCK8 reagent in liver cancer cells overexpressed or knockdown HMGCR treated with RSL3, Fer‐1, RSL3, and Fer‐1. F) IF assay was performed to detect the location of HMGCR in HepG2 cells treated with RSL3, LPS and Nig, CHX and TNFα. Scale bar: 10 µm. G) Western blot assay was used to detect the location of HMGCR in HepG2 cells treated with RSL3, LPS, and Nig. Data are presented as mean ± S.D (*n* = 3), and the P value was calculated using two‐sided unpaired Student's t‐tests (B, E). **P < 0.01.

We next explored whether HMGCR is related to pyroptosis and ferroptosis. We first observed the cell morphology in liver cancer cells with overexpressed HMGCR after treatment with pyroptosis inducers and found that the number of cells undergoing pyroptosis was increased (**Figure** **2**A). Moreover, HMGCR overexpression increased the release of LDH by the LDH assay (Figure 2B). Conversely, knockdown of HMGCR significantly reduced the number of pyroptotic cells and LDH release (Figure 2A,B). We further detected the mRNA expression of pyroptosis‐associated genes and found that HMGCR knockdown decreased the mRNA expression of these genes in liver cancer cells, whereas HMGCR overexpression increased their expression (Figure S2A,B, Supporting Information). We also detected the expression of the proteins and found that HMGCR overexpression increased, but HMGCR knockdown downregulated the expression level of NLRP3 and GSDMD (Figure 2C,D).

We further examined the potential role of HMGCR in ferroptosis. As shown in Figure 2E, overexpression of HMGCR significantly inhibited cell death triggered by RSL3, while the knockdown of HMGCR increased RSL3‐induced cell death. We also found that overexpression of HMGCR increased the expression level of GPX4 (Figure 2C). Since HMGCR is a key rating‐enzyme of the mevalonate pathway, we hypothesized that the mevalonate metabolic pathway plays a role in the regulation of pyroptosis in cancer cells. To test this hypothesis, we measured LDH release in HMGCR‐knockdown cells with and without the interference of mevalonate (MVA). The results showed no significant difference in LDH release after the addition of mevalonate, indicating that the mevalonate pathway does not have the ability to modulate pyroptosis (Figure S2C,D, Supporting Information). We then raised the possibility of whether the location of HMGCR in mitochondria or endoplasmic reticulum (ER) could determine the mode of cell death. As shown in Figure 2F and Figure S2E (Supporting Information), HMGCR was primarily located in mitochondria after treatment with RSL3, while HMGCR was predominantly located in ER after treatment with pyroptosis inducers. We further extracted the mitochondria and cytosolic fractions and found that the expression of HMGCR was altered after treatment with RSL3, LPS, and Nig (Figure 2G). Moreover, we utilized an IP‐MS assay to search for proteins that interact with HMGCR in HepG2 cells subjected to various treatments, including RSL3, LPS and Nig. Through analysis of changes in protein binding intensity and subcellular localization, our attention was drawn to SLC16A3 (solute carrier family 16 member 3) and RPL27 (ribosomal protein L27) (Table S1, Supporting Information). We further validated this with an IP experiment and found that HMGCR was more strongly bonded to SLC16A3 in cells treated with RSL3 compared to LPS and Nig, while the interaction between HMGCR and RPL27 was stronger in cells treated with LPS and Nig compared to RSL3 (Figure S2F, Supporting Information). This suggests that HMGCR promotes pyroptosis in the endoplasmic reticulum and inhibits ferroptosis in mitochondria.

### 2.3 BRCC36 Interacts with HMGCR

**Figure 3:**
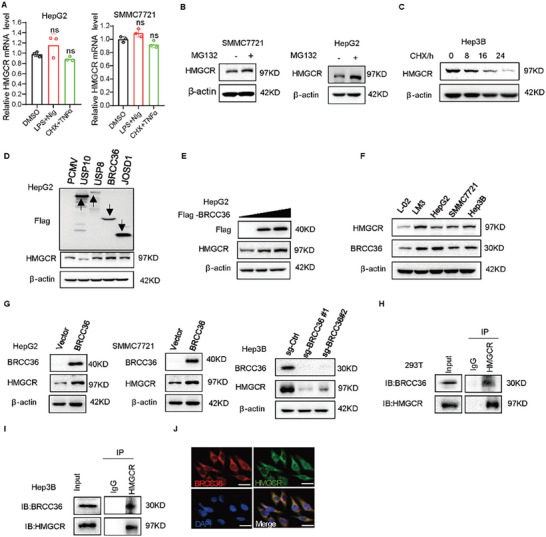
BRCC36 interacts with HMGCR. A) The expression of HMGCR mRNA was detected by using the qPCR assay in HepG2 cells treated with pyroptosis inducers LPS and Nig, CHX and TNFα. Liver cancer cells were treated with MG132 B) or CHX C) for the indicated time, and a western blot assay was performed to detect the expression levels of BRCC36 and HMGCR. D) HepG2 cells were transfected with USP10, USP8, BRCC36, and JOSD1 plasmids, and the expression levels of Flag and HMGCR was detected by using western blot assay. E) HepG2 cells were transfected with the BRCC36 plasmid in a dose‐dependent manner, and the expression levels of Flag and HMGCR was detected by using western blot assay. F) Western blot assay was performed to detect the expression levels of BRCC36 and HMGCR in normal cells and cancer cells. G) The expression levels of BRCC36 and HMGCR in liver cancer cells with BRCC36 overexpressed or depleted by using western blot assay. H) 293T cells were transfected with the BRCC36 plasmid for 48 h, and co‐IP assays were performed to detect the interaction of BRCC36 and HMGCR. I) Co‐IP assays was performed to detect the interaction of BRCC36 and HMGCR in Hep3B cells. J) IF assay was performed to detect the location of BRCC36 and HMGCR in Hep3B cells. Scale bar: 20 µm. Data are presented as mean ± S.D (*n* = 3), and the P value was calculated using two‐sided unpaired Student's t‐tests (A). n.s., not significant.

We next determined whether the change of HMGCR expression was regulated at the transcriptional level or post‐translational level. The mRNA level of HMGCR was not significantly altered in LPS and Nig, CHX and TNFα treatments (**Figure** **3**A), indicating that the inducers mainly regulate the post‐translational level of HMGCR. HMGCR is easily susceptible to proteasomal degradation, which is regulated by ubiquitin E3 ligases.^[^ ^36^ ^]^ However, the deubiquitinases (DUBs) which maintain a high level of HMGCR protein are rarely reported. We first examined the degradation of the HMGCR protein through the ubiquitin‐proteasome system and found that the expression of HMGCR increased after treatment with MG132 (Z‐Leu‐Leu‐Leu‐al) (Figure 3B). Moreover, the expression of HMGCR was gradually degraded with cycloheximide (CHX) treatment (Figure 3C). To comprehensively identify specific DUBs, we transiently expressed a series of plasmids individually encoding 44 DUBs fused with the Flag protein in 293T cells, and found that USP8, USP10, BRCC36, and JOSD1 enhanced the HMGCR protein level (Figure S3A, Supporting Information). Next, we transfected these four candidates into HepG2 cells and found that BRCC36 significantly increased the expression of HMGCR protein (Figure 3D). BRCC36 (BRCA1‐BRCA2‐containing complex subunit 36) belongs to the JAMM/MPN family of zinc metalloproteases and plays an essential role in protein stabilization in the cytosol.^[^ ^37^ ^]^ We transfected the BRCC36 plasmid in a dose‐dependent manner and observed a gradual increase in the expression of HMGCR in HepG2 cells (Figure 3E). Subsequently, we found that the expression of HMGCR and BRCC36 was upregulated and positively correlated in liver cancer cell lines and in clinical sample (Figure 3F; Figure S3B, Supporting Information). The expression of HMGCR increased after overexpression of BRCC36 in HepG2 and SMMC7721 cells, but the expression of HMGCR decreased after knockout of BRCC36 in Hep3B and LM3 cells (Figure 3G; Figure S3C, Supporting Information), without affecting the HMGCR mRNA level (Figure S3D, Supporting Information). Plasmids encoding Flag‐BRCC36 and HMGCR were co‐expressed in 293T cells, and coimmunoprecipitation (co‐IP) assays were performed. Figure 3H shows that HMGCR could interact with BRCC36. We further found an interaction between endogenous HMGCR and BRCC36 in Hep3B cells (Figure 3I). Furthermore, the immunofluorescence (IF) assay demonstrated the interaction between BRCC36 and HMGCR (Figure 3J). These results suggest that BRCC36 can interact with HMGCR.

### 2.4 BRCC36 Stabilizes HMGCR Protein through Deubiquitination

**Figure 4:**
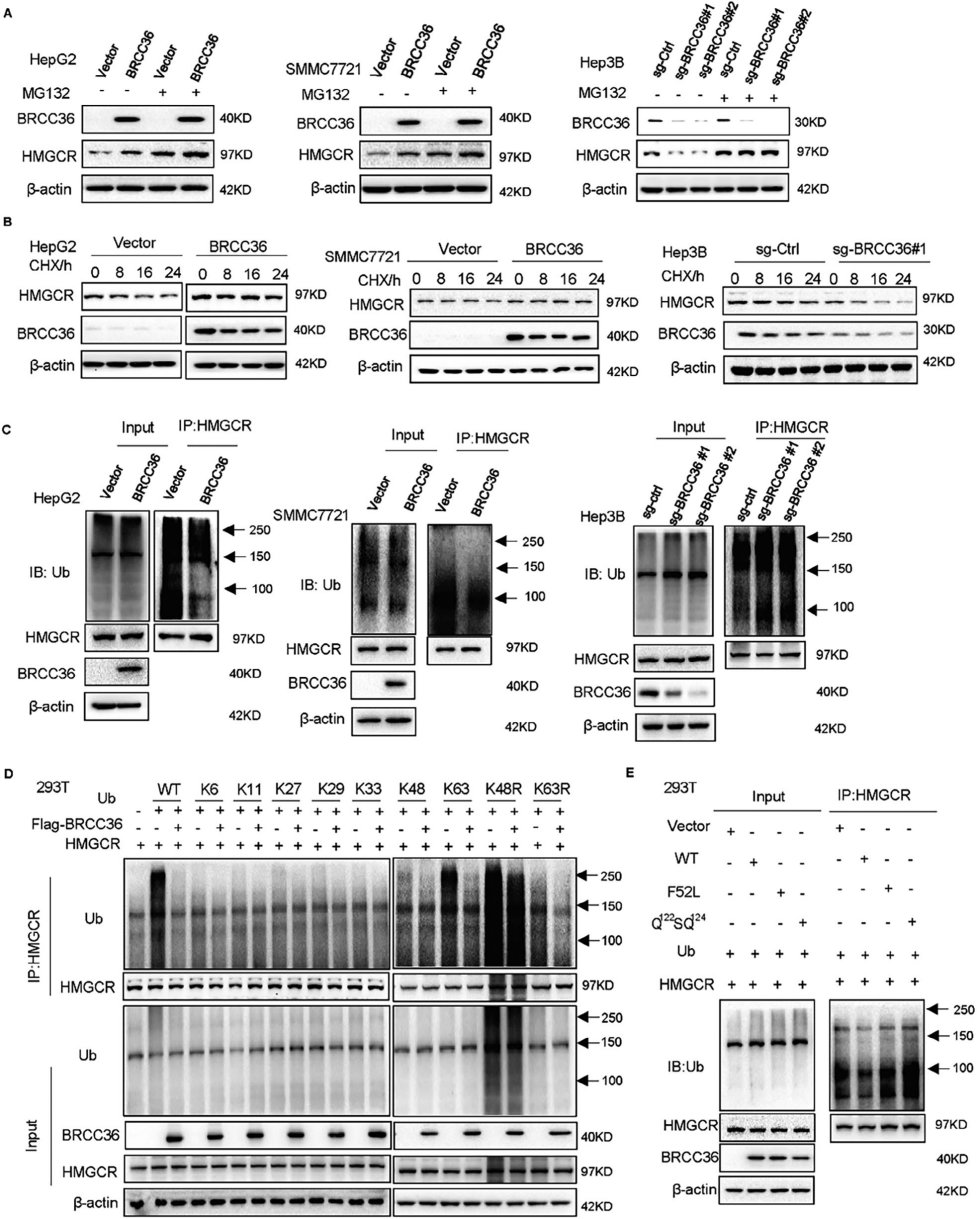
BRCC36 stabilizes the HMGCR protein through deubiquitination. The overexpression or depletion of BRCC36 cells was treated with MG132 A) or CHX B). The expression levels of BRCC36 and HMGCR was detected by using western blot assay. C) Liver cancer cells overexpressing or depleting BRCC36 were treated with MG132 for 12 h, and the ubiquitinated HMGCR was detected by using co‐IP assay. 293T cells were transfected with plasmids containing various polyubiquitin chains D), and transfected with BRCC36 wildtype or mutant plasmids E) for 48 h, and the ubiquitinated HMGCR was detected using co‐IP assay.

We further explore the efficacy of BRCC36 in delaying the degradation of HMGCR. We found that the level of HMGCR protein increased after overexpression or depletion of BRCC36 in cells treated with MG132 (**Figure** **4**A). Figure 4B shows that BRCC36 overexpression significantly inhibited the degradation of HMGCR in HepG2 and SMMC7721 cells treated with CHX, while HMGCR protein was markedly degraded after depletion of BRCC36 in Hep3B cells treated with CHX.

To examine whether BRCC36 blocks the ubiquitination of HMGCR, we detected the ubiquitination level of HMGCR. The ubiquitin level of HMGCR was decreased after overexpression of BRCC36 and increased after its depletion (Figure 4C), suggesting that BRCC36 can reduce the ubiquitination level of HMGCR through deubiquitination. We also detected the ubiquitin level of HMGCR in HepG2 cells treated with 25‐HC (25‐hydroxycholesterol, to stimulate the ubiquitination of HMGCR) and found that the ubiquitination level of HMGCR was decreased after overexpressing BRCC36 (Figure S4A, Supporting Information). Moreover, to determine which type of ubiquitin linkage of polyubiquitin chains on HMGCR is mediated by BRCC36, we transfected plasmids of polyubiquitin chains conjugated to the lysine 6 site (K6), K11 site, K27 site, K29 site, K33 site, K48 site, K63 site, and ubiquitin mutants K48R and K63R. Figure 4D shows that overexpression of BRCC36 reduced K63‐linked ubiquitination of HMGCR without affecting other types of linkages, indicating that BRCC36 primarily removes K63‐linked polyubiquitin chains of HMGCR. Moreover, we verified the binding sites of BRCC36 and HMGCR through point mutation experiments. We co‐transfected 293T cells with WT‐BRCC36, BRCC36‐F52L, and BRCC36‐Q^122^SQ^124^ mutant plasmids. Figure 4E shows that WT‐BRCC36, instead of BRCC36‐Q^122^SQ^124^, decreased the levels of polyubiquitinated HMGCR protein. These results suggest that BRCC36 directly deubiquitinates HMGCR.

### 2.5 BRCC36 Inhibits Ferroptosis and Promotes Pyroptosis

**Figure 5:**
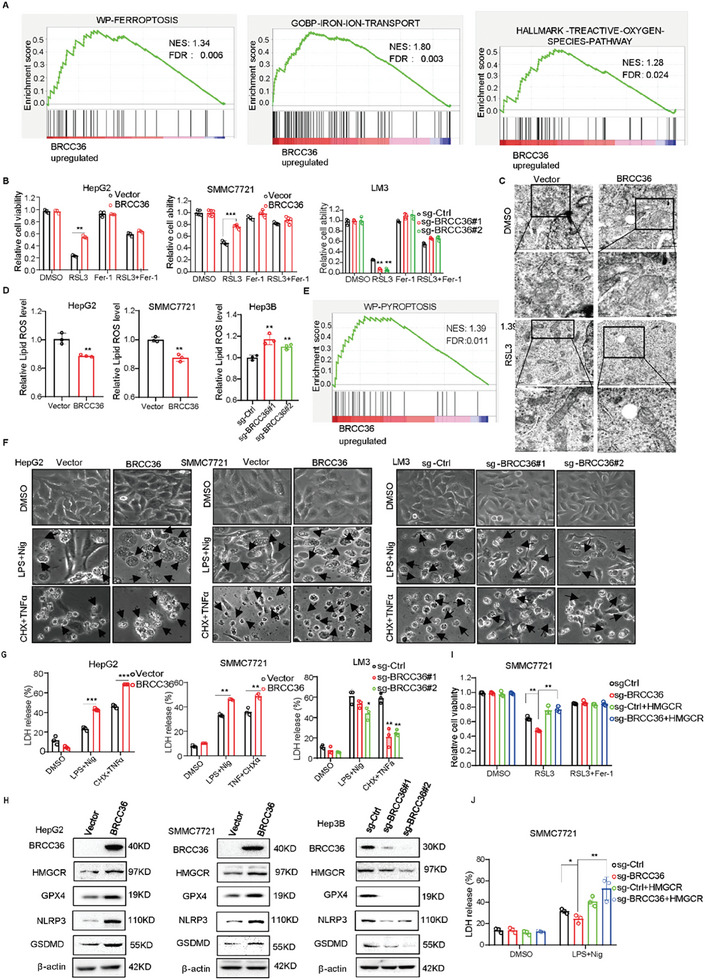
BRCC36 inhibits ferroptosis and promotes pyroptosis. A) The relationship between BRCC36 and the ferroptosis pathway was predicted by using GSEA in HCC. B) The liver cancer cells with BRCC36 overexpression or depletion were treated with RSL3, Fer‐1, RSL3 for 48 h, and the cell viability was measured by CCK8 reagent. C) Electron microscopy images of mitochondria in HepG2 cells treated with DMSO or RSL3. Scale bars,1 µm or 500 nm. D) The liver cancer cells overexpressing or depleting BRCC36 were treated with RSL3 for 24 h, and flow cytometry was performed to measure the level of lipid ROS. E) The relationship between BRCC36 and the pyroptosis pathway was predicted by using GSEA in HCC. Liver cancer cells overexpressed or depleted BRCC36 were treated with pyroptosis inducers LPS and Nig, CHX and TNFα. The pyroptotic morphology was observed F), black arrows represent pyroptotic cells, and LDH release by using LDH kit G). H) Western blot assay was performed to detect the expression levels of proteins associated with ferroptosis and pyroptosis in liver cancer cells with BRCC36 overexpression or depletion. I) The BRCC36‐depleted SMMC7721 cells were transfected with the HMGCR plasmid and treated with RSL3 or Fer‐1 for 48 h, and the cell viability was detected by CCK8 assay. J) The BRCC36‐depleted SMMC7721 cells were transfected with the HMGCR plasmid and treated with LPS and Nig, and the LDH release was measured by using LDH kit. Data are presented as mean ± S.D (*n* = 3), and the P value was calculated using two‐sided unpaired Student's t‐tests (B, C, G, I, J). *P < 0.05, **P < 0.01, ***P < 0.001.

To uncover the potential role of BRCC36 in ferroptosis, we first utilized GSEA (gene set enrichment analysis) to explore the effect of BRCC36 on ferroptosis in HCC. **Figure** **5**A shows significant differences in the pathway of “WP Ferroptosis”, “iron ion transport”, “reactive oxygen species pathway” between the high and low BRCC36 expression group, which suggested that may be involved in ferroptosis. We next found that the overexpression of BRCC36 inhibited RSL3‐induced cell death, while its knockout increased cell death, while this effect was subsequently rescued by the ferroptosis inhibitor fer‐1 (Figure 5B; Figure S5A, Supporting Information). Transmission electron microscopy analysis further revealed that HepG2 cells treated with RSL3 exhibited a typical ferroptotic morphology, characterized by shrunken mitochondria with condensed membranes. However, the overexpression of BRCC36 attenuated the change in mitochondrial morphology (Figure 5C). We also examined the effect of stable overexpression and knockdown of BRCC36 on lipid peroxidation, and found that BRCC36 overexpression reduced lipid peroxidation triggered by RSL3, whereas its depletion elevated levels of lipid ROS (Figure 5D). Moreover, BRCC36 overexpression upregulated the levels of ferroptosis suppressors HMGCR and GPX4, while those expressions decreased after the knockout of BRCC36 (Figure 5H). BRCC36 overexpression also rescued the low expression of HMGCR and GPX4 caused by RSL3 in HepG2 cells (Figure S5B, Supporting Information). These results indicated that BRCC36 inhibited ferroptosis.

Next, we explored the impact of BRCC36 on pyroptosis. First, we also used GSEA to analyze the correlation between BRCC36 expression and pyroptosis, and found that the pathway of “WP Pyroptosis” was enriched in the group with high BRCC36 expression (Figure 5E). We found that overexpression of BRCC36 increased the number of cells in a state of pyroptosis (Figure 5F) and LDH release (Figure 5F,G). Conversely, the knockout of BRCC36 significantly reduced the number of pyroptotic cells and LDH release (Figure 5F,G). The levels of pyroptosis regulators NLRP3 and GSDMD were upregulated in HCC cells with BRCC36 overexpression, while their expressions decreased after knockout of BRCC36 (Figure 5H).

**Figure 6:**
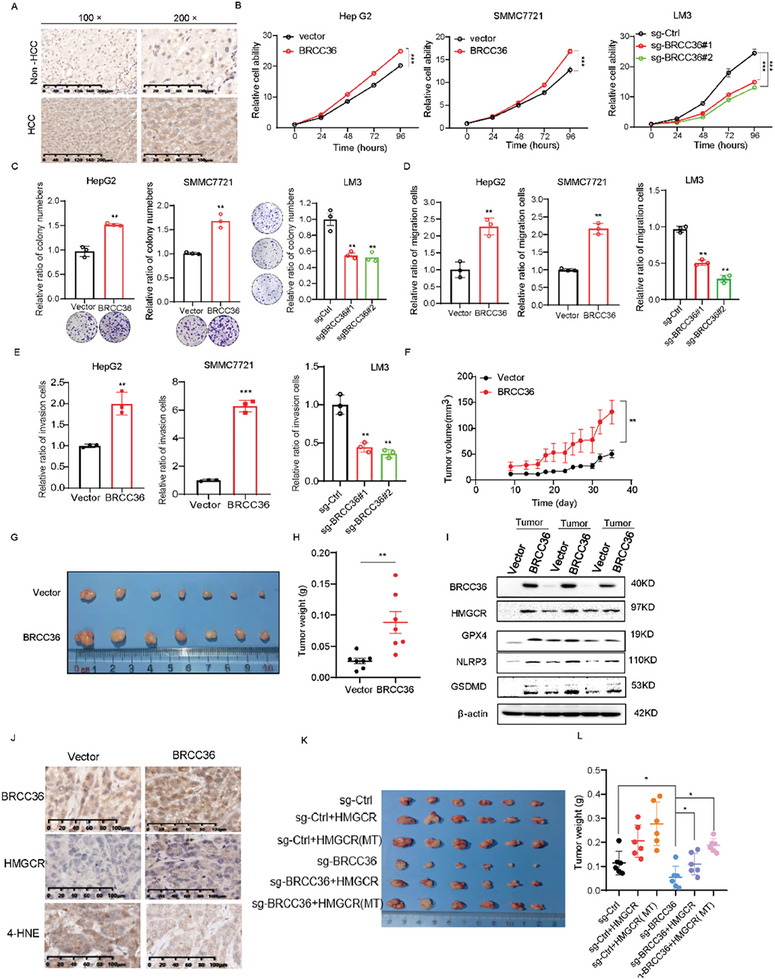
BRCC36 functions as an oncogene in hepatic carcinoma. A) The expression of BRCC36 in liver cancer tissues and paracancerous normal tissues by using IHC assay (magnification, ×100 scale bar = 200 µm; magnification, ×200 scale bar = 100 µm). B) The cell viability in HepG2, SMMC7721, and LM3 cells stably overexpressed or depleted of BRCC36. C) The colony formation ability in HepG2, SMMC7721, and LM3 cells stably overexpressed or depleted BRCC36. D) The cell migration ability in HepG2, SMMC7721 and LM3 cells overexpressed or depleted of BRCC36. E) The cell invasion ability in HepG2, SMMC7721 and LM3 cells with overexpressed or depleted of BRCC36. The xenograft model of tumor growth was established to evaluate the ability of SMMC7721 cells with stable BRCC36 overexpression. Dissected tumors F), tumor volume was monitored at the indicated times G), and weight H). I) The expression levels of BRCC36, HMGCR, GPX4, GSDMD, and NLRP3 in tumor samples. J) The expression levels of BRCC36, HMGCR, and 4‐HNE were detected by IHC assay in tumor samples. Scale bar, 100 µm. K) The xenograft model of tumor growth was established to evaluate the ability of BRCC36‐knockout cancer cells LM3 with re‐expressing wild‐type (WT) or mutant HMGCR (K248R). Dissected tumors (K), and weight L). Data are presented as mean ± S.D (*n* = 3), and the P value was calculated using two‐sided unpaired Student's t‐tests (B to E, H, L). Data are mean ± SEM (*n* = 6), and the P value was analyzed by one‐way ANOVA test followed by Turkey's multiple comparisons (F). *P < 0.05, **P < 0.01, ***P < 0.001.

Since HMGCR is a key suppressor of ferroptosis and a substrate of BRCC36, we hypothesized that HMGCR is involved in BRCC36‐mediated ferroptosis and pyroptosis. Notably, the re‐expression of HMGCR suppressed RSL3‐induced cell death and increased the LDH release in BRCC36‐knockout cancer cells (**Figure** **6**I,J; Figure S5E, Supporting Information). Moreover, overexpression of BRCC36 upregulated the expression of HMGCR and GPX4 in HepG2 cells treated with LPS and Nig (Figure S3C, Supporting Information). Furthermore, we extracted the mitochondria and cytosolic fractions in HepG2 cells with BRCC36 overexpression. We found that HMGCR expression was significantly upregulated in the cytosolic fractions following treatment with LPS and Nig, whereas HMGCR expression was increased upon treatment with RSL3 in the mitochondria fractions of BRCC36 overexpressed cells (Figure S3H, Supporting Information). It indicates that BRCC36 could regulate HMGCR expression to promote pyroptosis in the endoplasmic reticulum and inhibit ferroptosis in mitochondria. Therefore, these findings indicate that depletion of BRCC36 promotes ferroptosis and inhibits pyroptosis primarily by regulating HMGCR expression.

### 2.6 BRCC36 Functions as an Oncogene in Hepatic Carcinoma

BRCC36 plays a critical role in a variety of pathophysiological processes, such as DNA repair and inflammatory regulation. However, the effect of BRCC36 on hepatic carcinoma remains elusive. A Kaplan‐Meier analysis showed that elevated BRCC36 expression was associated with shorter overall survival compared to low BRCC36 expression (Figure S6A, Supporting Information). TCGA database analysis indicated that the mRNA level of BRCC36 was increased in HCC (Figure S6B, Supporting Information). Moreover, immunohistochemical (IHC) analysis of tissue samples showed that the BRCC36 expression was significantly upregulated in liver cancer tissue (Figure 6A). Moreover, BRCC36 was upregulated in liver cancer cell lines and in clinical liver cancer patients (Figure 3F; Figure S3B, Supporting Information). These findings suggest that the expression level of BRCC36 is positively correlated with HCC progression.

We further examine the physiological role of BRCC36 in HCC. We found that BRCC36 overexpression significantly promoted cell growth and the ability of colony formation, while its knockout resulted in the inhibition of cell growth and colony formation (Figure 6B,C). Moreover, we found that overexpression of BRCC36 significantly increased cell migration and invasion, while knockout of BRCC36 suppressed these processes (Figure 6D,E; Figure S6C,D, Supporting Information).

To further examine whether BRCC36 overexpression promotes HCC cell growth in vivo, an SMMC7721 xenograft model was established. BRCC36 overexpression significantly accelerated tumor growth, as demonstrated by the increase in tumor volumes and weights (Figure 6F–H). We further detected the expression of ferroptosis‐associated proteins (HMGCR, GPX4) and pyroptosis‐associated proteins (such as NLRP3, GSDMD) in tumor tissues by using immunohistochemistry or western blot. We found that BRCC36 overexpression upregulated the expression of GPX4 and HMGCR, while decreasing the levels of 4‐HNE (4‐hydroxy‐2‐noneal), a biomarker of ferroptosis (Figure 6I,J), indicating BRCC36 inhibits ferroptosis in HCC. We also found that overexpression of BRCC36 increased the expression of NLRP3 and GSDMD (Figure 6I; Figure S6F, Supporting Information). Moreover, we constructed the C57BL/6 subcutaneous transplanted tumor model by using Hepa1‐6 with Brcc36 knockdown. We found that the knockdown of Brcc36 significantly inhibited tumor growth, as demonstrated by the significantly decreased tumor volumes and weights, indicating that Brcc36 hinders the proliferation of hepatocellular carcinoma cells (Figure S6F–H, Supporting Information). We further established the xenograft model by re‐expressing wild‐type (WT) or mutant HMGCR (K248R) in BRCC36‐knockout cancer cells. We found that overexpression of HMGCR or mutant HMGCR significantly abrogated the tumor growth suppression in BRCC36‐knockout tumors, indicating that the oncogenic effect of BRCC36 is dependent on HMGCR (Figure 6K,L; Figure S6I–K, Supporting Information). Interestingly, we analyzed the relationship between the overall survival rate and the expression of HMGCR and BRCC36. The results showed a negative correlation between the expression levels of BRCC36 and HMGCR in patient prognosis. Liver cancer patients with high expression of both BRCC36 and HMGCR had the worst prognosis (Figure S6L, Supporting Information). This suggests that BRCC36 and HMGCR are negatively correlated with patient prognosis and could potentially serve as prognostic factors in HCC.

### 2.7 Thiolutin Weakens the Interaction between BRCC36 and HMGCR and Inhibits Tumor Growth in HCC

**Figure 7:**
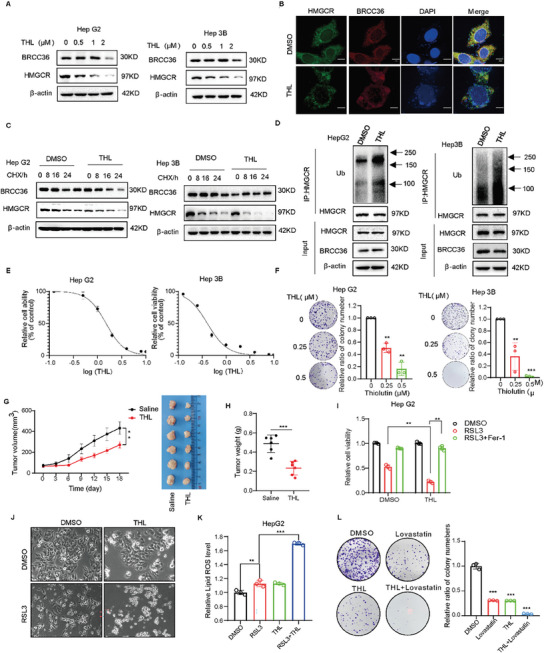
Thiolutin weakens the interaction between BRCC36 and HMGCR and inhibits tumor growth in HCC. A) The expression levels of BRCC36 and HMGCR proteins was detected by a western blot assay in HepG2 and Hep3B cells treated with the indicated concentrations of THL for 24 h. B) Immunofluorescence of BRCC36 and HMGCR in HepG2 cells after THL treatment for 24 h. Scale bar, 5 µm. C) The expression levels of BRCC36 and HMGCR proteins in HepG2 and Hep3B cells treated with THL, DMSO, or CHX for the indicate time. D) HepG2 and Hep3B cells were pretreated with THL or DMSO for 24 h, and then incubated with MG132 for an additional 12 h. The ubiquitinated HMGCR was detected by IP assay. E) The cell viability was detected using the CCK8 reagent in HepG2 and Hep3B cells treated with the indicated concentrations of THL for 48 h. F) The colony formation ability in HepG2 and Hep3B cells treated with 0.25 or 0.5 µm THL. G) Tumor volume was measured every three days to determine the growth rate of THL‐treated xenografts. And the tumor weight was measured H). I) The cell viability in HepG2 cells treated with THL, RSL3, or Fer‐1 for 48 h. J) Representative phase‐contrast images of HepG2 cells treated with THL or RSL3 for 24 h. K) Flow cytometry was performed to measure the lipid ROS level in HepG2 cells treated with THL or RSL3 for 24 h. L) The clonogenic ability of HepG2 cells treated with THL (0.5 µm) and lovastatin (5 µm). Data are presented as mean ± S.D (*n* = 3), and the P value was analyzed by a two‐tailed unpaired Student's t‐test (F, H, I, K, L). Data are mean ± SEM (*n* = 6), and the P value was analyzed by one‐way ANOVA test followed by Turkey's multiple comparisons G). *P < 0.05, **P < 0.01, ***P < 0.001.

Thiolutin (THL), a disulfide‐containing antibiotic synthesized by Streptomyces, has been identified as an inhibitor of JAMM metalloprotease BRCC36.^[^ ^38^ ^]^ We first found that THL inhibited the enzyme activity of BRCC36 through molecular docking, and significantly inhibited the expression of BRCC36 and HMGCR proteins in a dose‐dependent manner (**Figure** **7**A, and Figure S7A,B, Supporting Information), but had no effect on HMGCR mRNA expression (Figure S7C, Supporting Information). We further performed an IF assay to determine the interaction between HMGCR and BRCC36 in HepG2 cells treated with THL, and found that the interaction was attenuated (Figure 7B). We then explored whether THL could inhibit the deubiquitinase activity of BRCC36 against HMGCR. Figure 7C shows that THL treatment significantly reduced the half‐life of the HMGCR protein. Moreover, the ubiquitin level of HMGCR was increased after THL treatment (Figure 7D), indicating that the ability of BRCC36 to deubiquitinate HMGCR was attenuated. These findings suggest that THL accelerates the degradation of HMGCR through the ubiquitin‐proteasome system.

Next, we examined the effect of THL on the proliferation of HCC. It was found that THL significantly decreased cell proliferation in a dose‐dependent manner (Figure 7E), and the colony formation assay further confirmed that THL inhibited cell proliferation (Figure 7F). Moreover, HMGCR overexpression inhibited THL‐induced cell death, indicating that the anti‐cancer mechanism of THL is related to the decreased expression of HMGCR (Figure S7D, Supporting Information). We established a subcutaneous xenograft model to elucidate the impact of THL on HCC in vivo. As shown in Figure 7G, THL significantly decreased the volume and weight of the tumor. Additionally, we found that THL significantly elevated RSL3‐induced cell death (Figure 7H,I) and RSL3‐triggered lipid peroxidation (Figure 7J), indicating that THL treatment could stimulate ferroptosis. These findings suggest that the inhibition of BRCC36 by THL provides a novel and effective therapeutic approach for liver cancer treatment by promoting ferroptosis. THL inhibited the LDH release induced by LPS and Nigericin (Figure S7E, Supporting Information), indicating that THL might suppress pyroptosis. Moreover, the combination treatment with THL and the HMGCR inhibitor lovastatin significantly reduced the colony‐forming ability of HepG2 cancer cells (Figure 7K). These findings suggest that THL inhibits tumor growth in HCC, implying that BRCC36 could be a new and promising therapeutic target for liver cancer treatment.

## 3 Discussion

**Figure 8:**
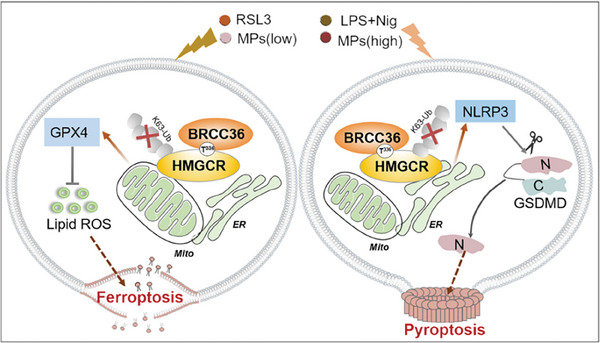
BRCC36 deubiquitinates HMGCR to regulate the interplay between ferroptosis and pyroptosis. BRCC36 stabilizes the HMGCR protein by eliminating the K63 polyubiquitin chain. HMGCR is primarily located in the mitochondria when cells are exposed to the ferroptosis inducer RSL3 or low concentration of MPs. It prevents the accumulation of lipid peroxides by modulating GPX4 activity, thereby preventing ferroptosis. When cells were exposed to LPS and Nig, or high concentrations of MPs, HMGCR was predominantly located in the endoplasmic reticulum. This localization had an impact on the assembly of GSDMD‐N protein on the membrane and subsequently led to pyroptosis.

The lytic forms of cell death, including pyroptosis and ferroptosis, trigger robust inflammatory responses, which contribute to antitumor immunity by causing cell membrane disruption and the release of cellular components, such as damage‐associated molecular patterns (DAMPs). For instance, the high‐mobility group box 1 (HMGB1), which is considered a major pro‐inflammatory DAMP, is released by cancer cells undergoing ferroptosis or pyroptosis, thereby stimulating inflammatory responses.^[^ ^39^, ^40^ ^]^ Numerous studies have shown that ferroptosis contributes to inflammation or immunogenicity. However, comparing ferroptosis to pyroptosis, necroptosis, or apoptosis in general can be challenging.^[^ ^41^ ^]^ In this study, we first found that MPs or LPS induced ferroptosis and pyroptosis at different concentrations, and ferroptosis inhibitor Fer‐1 or Lip‐1 significantly increased cell death triggered by pyroptosis inducers. Furthermore, knockdown of GSDMD/GSDME enhanced cell death induced by RSL3 or ML162. In order to investigate the relationship between ferroptosis and pyroptosis, we conducted a more detailed analysis of the protein levels of key regulators of ferroptosis and pyroptosis in liver cancers treated with inducers of ferroptosis and pyroptosis to explore the interaction between ferroptosis and pyroptosis. Our findings indicate that there exists a negative crosstalk between ferroptosis and pyroptosis (**Figure** **8**).

The interaction and switch between different forms of cell death are regulated by specific factors. Ferroptosis is a form of cell death that depends on autophagy and relies on lysosomes. Nuclear receptor coactivator 4 (NCOA4) interacts with FTH (ferritin heavy chain) to mediate the autophagic degradation of ferritin, and contributes to an increase in intracellular free iron and erastin‐induced ferroptosis.^[^ ^42^, ^43^ ^]^ Overexpression of LAMP2A (lysosome‐associated membrane‐protein type 2A) promotes GPX4 degradation by CMA (chaperone‐mediated autophagy), ultimately leading to ferroptosis.^[^ ^14^ ^]^ Several pieces of evidence document that crosstalk exists between necroptosis and pyroptosis through the executioner MLKL (mixed‐lineage kinase domain‐like protein).^[^ ^44^, ^45^ ^]^ Moreover, ZBP1 (Z‐DNA‐binding protein) is crucial for the activation of intricate interplay between pyroptosis, apoptosis, and necroptosis after influenza A virus (IAV) infection.^[^ ^46^ ^]^ Research also shows that caspase‐8 mediates the switch between apoptosis, necroptosis, and pyroptosis.^[^ ^47^ ^]^ Our research highlights that HMGCR could play a crucial role in the correlation between ferroptosis and pyroptosis. Previous studies have reported that HMGCR suppresses ferroptosis by interfering with GPX4 biosynthesis. Here, we found that HMGCR promotes pyroptosis, likely by regulating the NLRP3‐caspase‐1‐GSDMD pathway. Interestingly, we found that HMGCR was more strongly bonded to SLC16A3 in cells treated with RSL3 compared to LPS and Nig, while the interaction between HMGCR and RPL27 was stronger in cells treated with LPS and Nig compared to RSL3. This suggests that the subcellular localization of HMGCR in ER or mitochondria could affect ferroptosis and pyroptosis.

Protein ubiquitination and deubiquitination play indispensable roles in post‐translational modification by regulating substrate degradation, localization, and signal transduction. DUBs are powerful regulators of cell fate that can modulate multiple types of PCD. For example, the deubiquitinase OTUB1 is abnormally upregulated in cancer cells and promotes tumor development to suppress ferroptosis by stabilizing the cystine transporter SLC7A11, which uptakes cystine and exports glutamate.^[^ ^48^ ^]^ DUBs promote or suppress different types of PCD, which mainly depend on downstream signaling. The tumor suppressor BRCA1‐associated protein 1 (BAP1) represses cystine uptake by inhibiting SLC7A11 expression and promotes ferroptosis.^[^ ^49^ ^]^ Moreover, BAP1 induces apoptosis by binding to the 14‐3‐3 protein or suppresses apoptosis by stabilizing liver kinase B1 (LKB1). Here, we found that BRCC36 acts as a DUB to stabilize HMGCR. In HCC, BRCC36 functions as an oncogene, promoting cell proliferation, migration, and tumor formation. We also found that BRCC36 inhibits ferroptosis and regulates pyroptosis, which is dependent on HMGCR expression. It has been reported that severe and acute pyroptosis can contribute to tumor suppression, while chronic and spontaneous pyroptosis can promote tumor growth.^[^ ^50^ ^]^ BRCC36 overexpression upregulates GSDMD expression and induces chronic pyroptosis, both of which deteriorate the tumor microenvironment (TME) over time and accelerate tumor growth, but this needs to be further studied.

Furthermore, THL could inhibit the activity of the BRCC36 enzyme, reduce HMGCR protein levels, and suppress tumor growth. It reveals that BRCC36 could identify potential therapeutic targets for HCC treatment. THL's anti‐cancer effect may either promote ferroptosis or reduce pyroptosis, depending on the specific drug combination used. THL, for example, triggers cell death in response to RSL3. THL may potentially regulate cell death by controlling the localization of HMGCR in the endoplasmic reticulum and mitochondria, but further research is required. Interestingly, we also found that BRCC36 and HMGCR were highly expressed in HCC and cancer cells, and the high expression of BRCC36 and HMGCR was associated with shorter survival in patients. Furthermore, the combination treatment with THL and lovastatin reduced cell proliferation, indicating that the combination of HMGCR inhibitors, such as statins, and the BCC36 inhibitor THL could become a new therapeutic strategy for patients.

In summary, we explored the potential interactions between ferroptosis and pyroptosis in liver cancer cells. We discovered a mutually antagonistic interplay between ferroptosis and pyroptosis, and HMGCR promoted pyroptosis in the endoplasmic reticulum while inhibiting ferroptosis in the mitochondria. Furthermore, BRCC36 promoted cell proliferation, migration, and invasion, while inhibiting ferroptosis and inducing pyroptosis in liver cancer cells. Thiolutin triggered ferroptosis in liver cancer cells by targeting BRCC36, thereby inhibiting the progression of HCC. A comprehensive understanding of the differences and crosstalk between these cell death pathways will provide insights into how to target them for cancer therapy.

## 4 Experimental Section

### Cell Culture

Human 293T cells, the liver cancer cell lines Hep3B, HepG2, LM3, SMMC7721, and the human fetal hepatocyte line L‐02 were obtained from the Cancer Research Institute of Central South University. All cells were cultured in DMEM (Gibco, USA) medium supplemented with 10% bovine calf serum (BCS) (Sigma) and 1% penicillin and streptomycin, and were maintained at 37 °C with 5% CO2.

### Human Tissues

All clinical tumor tissue samples were obtained from patients at Xiangya Hospital of Central South University. The study protocol was approved by the Institutional Ethics Review Board of Xiangya Hospital, Central South University. Fresh samples were either pretreated with liquid nitrogen or stored at −80 °C until protein extraction, or were subjected to formalin fixation and embedded in paraffin for use in procedures such as immunohistochemistry assays. The number of human samples used were HCCP1486409, HCCP1389953, HCCP1243009, HCCP1390993, HCCP1158874, HCCP1346078, HCCP1482056, HCCP1486385, HCCP1248152.

### Antibodies and Reagents

The applied antibodies were as follows: anti‐BRCC36 (15391‐1‐AP), anti‐Ubiquitin (10201‐2‐AP), anti‐NLRP3 (19771‐1‐AP), and anti‐GSDMD (20770‐1‐AP) were purchased from Proteintech (Rosemont, Illinois, USA); anti‐HMGCR (sc‐271595) was purchased from Santa Cruz (Dallas, Texas, USA); anti‐cleaved‐GSDMD (#36 425), anti‐BRCC36 (#18215S), and Normal Rabbit IgG (#2729) were purchased from Cell Signaling Technology; anti‐HMGCR (ab174830), anti‐cleaved caspase‐1 (ab207802), and anti‐4‐Hydroxynonenal (ab46545) were purchased from Abcam. Anti‐β‐actin (A5441) and anti‐Flag (F1804) were purchased from Sigma‐Aldrich. Anti‐HMGCR (A19063) was purchased from ABclonal. The reagents used in the experiment were as follows: RSL3 (S8155) was obtained from Selleck; Ferrostatin‐1 (SML0583) and Lithium phosphorus sulfide (LPS) (L2630) were purchased from Sigma‐Aldrich; Thiolutin (THL) and 25‐hydroxycholesterol were obtained from APExBIO.

### Plasmids and Transfection

HMGCR and BRCC36 plasmids were purchased from Vigene Biosciences. The stable overexpression plasmid for HMGCR or BRCC36 was established by subcloning cDNA encoding the full open reading frames (ORFs) into the pLVX‐EF1α‐IRES‐Puro vector (catalog No. 631 988; Clontech). The MYC‐Ub‐WT, ‐K6, ‐K11, ‐K27, ‐K29, ‐K33, ‐K48, ‐K63, ‐K48R, and ‐K63R constructs were provided by Professor Pinglong Xu from the Life Sciences Institute and Innovation Center for Cell Signaling Network at Zhejiang University, China. The sh‐HMGCR targeting regions, CAAGTTATTACCCTAAGTTTA (sh‐HMGCR #1) and TATAGCTGGACGCAACCTTTA (sh‐HMGCR #2) were inserted into pLVX‐shRNA1. The sg‐BRCC36 targeting regions of CAAAGCCTTGATCCATCATC (sg‐BRCC36#1) and ACAAGCCATGTACCAGATGA (sg‐BRCC36#2) were inserted into the lent guide‐Puro. All constructs were confirmed by DNA sequencing. Plasmid transfection using Neofect DNA transfection reagent was performed following the instructions provided by the manufacturer.

### Transwell Assay

Cells were seeded into the transwell, which was either precoated with Matrigel or left uncoated. The bottom chambers were filled with 600 µL of DMEM medium supplemented with 10% BCS. After incubation at 37 °C with 5% CO2 for 24 or 48 h, the upper chambers were fixed with methanol for 30 min and stained with 0.5% crystal violet for 1 h. The migratory or invasive cells were photographed using an inverted microscope.

### LDH Release Assay

Cells were cultured in 96‐well plates and treated with the indicated reagents. After completing the treatment, the supernatants were collected, and the levels of lactate dehydrogenase (LDH) were measured using the LDH Cytotoxicity Assay Kit purchased from Beyotime according to the manufacturer's instructions.

### Western Blot Analysis and Coimmunoprecipitation (Co‐IP) Assay

Cells were harvested and washed with PBS and then lysed in immunoprecipitation (IP) lysis buffer supplemented with a protease inhibitor cocktail (Bimake) on ice for 2 h. After centrifugation at 13 000 rpm, 4 °C for 15 min, the protein concentrations were measured using the BCA protein assay kit (Thermo Scientific). Samples were separated by SDS‐PAGE and transferred to PVDF membranes, followed by blocking with 10% skim milk. The membrane was then incubated with a primary antibody at 4 °C overnight. The next day, the membrane was incubated with HRP‐conjugated secondary antibodies at room temperature for 1 h. All membranes were visualized using the ChemiDox XRS+ image‐forming system (Bio‐Rad).

For the Co‐IP assay, the proteins (1 mg) were gently rotated in an IP lysis buffer‐containing cocktail and incubated with 2 µg of primary antibody at 4 °C overnight. The next day, a magnetic bead (ThermoFisher) was added to collect the precipitated protein complex using a magnetic rack. Then the beads were washed three times with cold PB or IP lysis buffer. Samples were then boiled at 100 °C for 5 min to release the bound protein. Total protein (20 µg) was used as input control. Samples were analyzed by a Western blot assay.

### Cell Viability Assay

Cells were cultured in 96‐well plates and treated with the indicated reagents. At the end of the treatment, 10 µL of cell counting kit‐8 (CCK‐8) reagent (Bimake) was added to each well and incubated for 2 h. At the end of this incubation, the absorbance at 450 nm was measured using a multiple‐well plate reader (BioTek).

### Colony Formation Assay

Cells (500 cells/well) were seeded in 6‐well plates and then treated with the specified concentrations of THL or not. After 14 days, cells were fixed with methanol, stained with 0.5% crystal violet, and the colonies were counted using ImageJ software.

### Transmission Electron Microscopy

Cells were cultured in 6‐well plates. After being treated with the indicated reagents for 24 h, cells were harvested and fixed with 1 mL of fixative. Subsequently, images were acquired using a transmission electron microscope (Hitachi; HT7700).

### Measurement of Lipid ROS

Cells were cultured and treated with the indicated reagents. At the end of the treatment, cells were harvested and resuspended in 1 mL of DMEM medium supplemented with 10% BCS and a 10 µm C11 BODIPY probe (Thermo Fisher Scientific; cat #D3861). Next, the samples were incubated for 30 min at 37 °C with 5% CO2 in the dark and then washed with PBS. Samples were analyzed using a flow cytometer (Fortessa, BD Biosciences) with a fluorescein isothiocyanate (FITC) green channel and a Texas red channel.

### Immunofluorescence Microscopy

Cells were cultured in 24‐well plates covered with glass overnight at 37 °C with 5% CO2 for 24 h, and then treated with the indicated reagents or not. Cells were fixed with methanol and then permeabilized with 0.5% Triton X‐100. After being blocked by 5% donkey serum, the cells were incubated with primary antibodies at 4 °C. Cells were incubated with secondary antibodies conjugated to Alexa Fluor 488 or Alexa Fluor 596 (Invitrogen) for 1 h at room temperature. The nuclei were stained with DAPI for 10 min. The images were obtained using a confocal microscope (Zeiss, Germany).

### Animal Experiments

The Hunan SJA Laboratory Animal Co., Ltd. supplied female nude mice at the age of 5 weeks. The project was approved by the ethics committee of the Cancer Research Institute of Central South University, and the approval number for animal experiments is 2023‐KT001. To establish the xenograft tumor models, SMMC7721, HepG2, or Heap1‐6 cells in 0.1 mL of PBS were subcutaneously injected into BALB/c or C57 mice (with a mean age of 5 weeks). After 7–10 days, the tumor volume and mice weight were measured every 3 days until sacrifice. After 7–10 days, the tumor volume and mice weight were measured every 3 days until sacrifice. To elucidate whether the oncogenic effect of BRCC36 was dependent on HMGCR, BRCC36‐knockout LM3 cells were subcutaneously injected, re‐expressing either wild‐type (WT) or mutant HMGCR, into BALB/c mice (mean age of 5 weeks) using 0.1 mL of PBS. After 10 days, tumor volume and mice weight were measured every 3 days until sacrifice. To assess the impact of THL in vivo, when the tumor sizes reached 100 mm3, the mice were administered with saline and THL (0.75 mg kg^−1^) every 3 days by intraperitoneal injection. Tumor volume and body weight were measured every three days. In the end, all mice were sacrificed, and the tumors were collected for further immunohistochemical staining or Western blot analysis.

### Statistical Analysis

Except for the nude mouse experiments, data was obtained from at least three independent experiments and expressed as mean ± SD. Statistical significance (P‐values) was calculated using two‐sided unpaired Student's t‐tests and two‐way analysis of variance (ANOVA) with GraphPad Prism 8.0 (GraphPad Software, Inc.). In these statistical results, ns is considered nonsignificant (*P* > 0.05), * indicates *P* < 0.05, ** indicates *P* < 0.01, and *** denotes *P* < 0.001.

## Conflict of Interest

The authors declare no conflict of interest.

## Author Contributions

Y.‐G.T. and H.‐Y.W. conceived and designed the manuscript. H.‐Y.W. performed the experiments and wrote the draft manuscript. L.S., C.‐R.L., N.L., Y.L., T.‐N.T., X.‐T.P., H.‐l.L, and J.T. conducted the data analysis. S.L. and D.‐S.X. gave insightful suggestions and comments on the outline of this manuscript; Y.‐G.T. revised and submitted this manuscript on behalf of other authors.

## Supporting information

Supporting Information

## Data Availability

Data sharing is not applicable to this article as no new data were created or analyzed in this study.
