# Supplementary material for: BRCC36 Deubiquitinates HMGCR to Regulate the Interplay Between Ferroptosis and Pyroptosis
Source: Adv Sci (Weinh). 2024 Jan 4;11(11):2304263. doi: 10.1002/advs.202304263 (PMC10953584; doi:10.1002/advs.202304263)
Supplement: Supplementary file 1 — Supporting Information [file ADVS-11-2304263-s001.pdf]

ADVANCED SCIENCE
Open Access

Supporting Information

for *Adv. Sci.*, DOI 10.1002/advs.202304263

BRCC36 Deubiquitinates HMGCR to Regulate the Interplay Between Ferroptosis and Pyroptosis

Haiyan Wang, Long Shu, Cairui Lv, Na Liu, Yao Long, Xintong Peng, Huli Ling, Tania Tao, Jun Tang, Yan Cheng, Shuang Liu, Desheng Xiao* and Yongguang Tao**

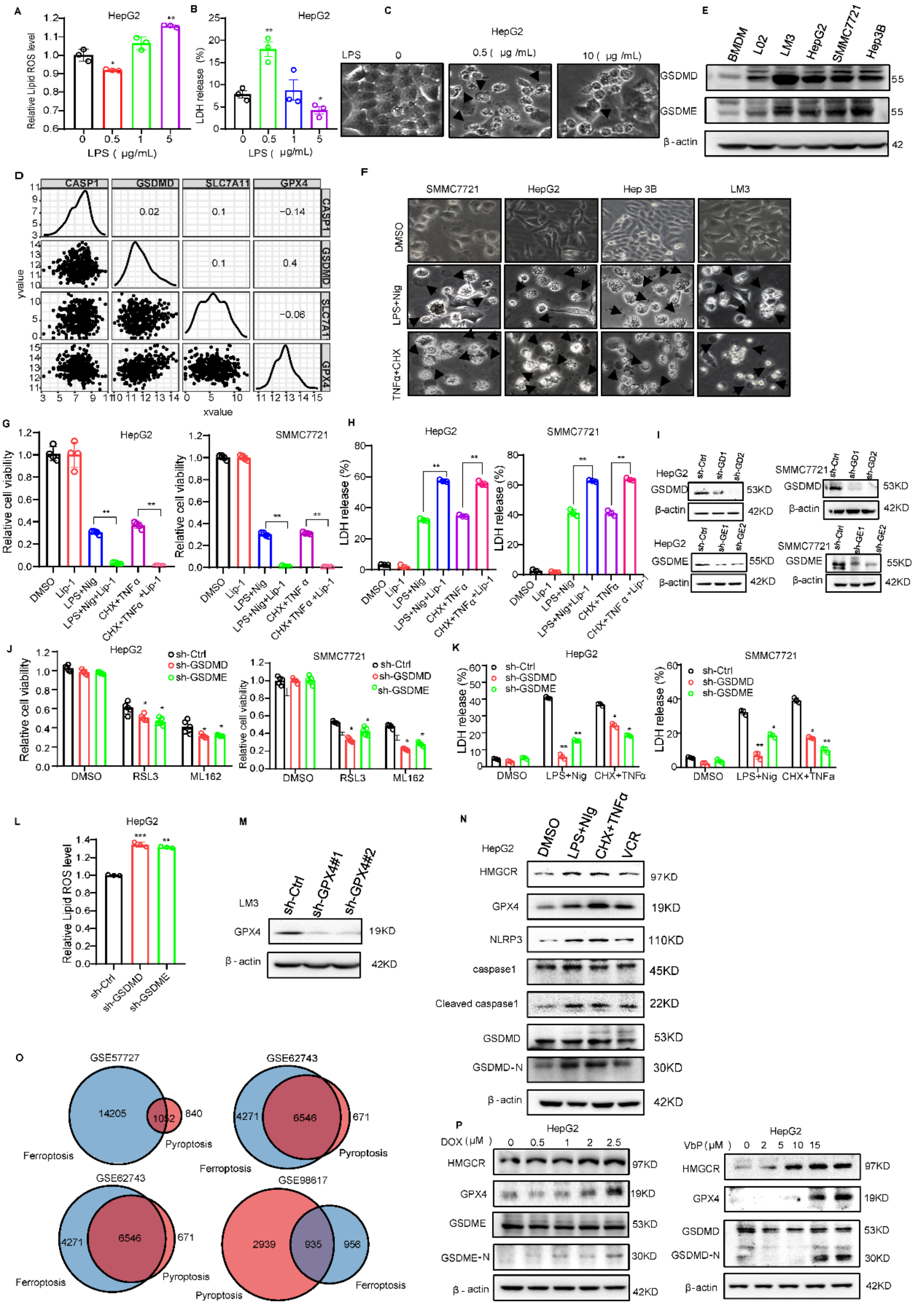

Fig. S1. HMGCR is a key regulator in regulating the correlation between ferroptosis and pyroptosis. HepG2 cells were treated with the indicated concentrations of LPS. Flow cytometry was performed to measure the relative lipid ROS level (A), and the release of LDH was measured by using LDH detection kits (B). The pyroptotic morphology was observed (C).

Black arrows represent pyroptotic cells. (D) The association between the key genes of ferroptosis (SLC7A11, GPX4) and pyroptosis (CASP1, GSDMD) were predicted by ssGSEA analysis using the TCGA database. (E) Western blot assay was performed to detect the expression of GSDMD and GSDME in HCC cancer lines LM3, SMMC7721, HepG2, Hep3B and normal cells L02, mouse bone marrow-derived macrophages (BMDMs). (F) The liver cancer cells were treated with pyroptosis inducers LPS and nigericin, CHX and TNFα. The pyroptotic morphology was observed. Black arrows represent pyroptotic cells. HepG2 and SMMC7721 cells were treated with pyroptosis inducers LPS and nigericin, CHX and TNFα, and Lip-1. Cell viability was detected by the CCK8 reagent (G), and LDH release was detected by the LDH kit (H). (I) The expression of GSDMD/GSDME was knocked down in HepG2 and SMMC7721 cells. Western blot assay was performed to detect the expression levels of GSDMD, and GSDME. HepG2 or SMMC7721 cells were knocked down GSDMD/GSDME. Analysis of the cell death after cells treated with RSL3 or ML162 (J) and LDH release after cells treated with pyroptosis inducers (K). (L) HepG2 and SMMC7721 cells with knockdown of GSDMD/GSDME were treated with ML162, and flow cytometry was performed to measure the relative lipid ROS level. (M) Western blot assay was performed to detect the expression of GPX4 in LM3 cells with knockdown of GPX4. (N) HepG2 cells were treated with LPS and nigericin, CHX and TNFα, apoptosis trigger vincristine (VCR), and western blot assay was performed to detect the expression levels of ferroptosis- and pyroptosis-associated proteins. (O) Venn diagrams showing the overlapping number of genes correlated with ferroptosis and pyroptosis score constructed by ssGSEA algorithm in GSE98617, GSE62743, GSE57727, and GSE78737 datasets, respectively. (P) HepG2 was treated with the indicated concentrations of pyroptosis-related drugs doxorubicin and talabostat. Western blot assay was performed to detect the expression levels of ferroptosis- and pyroptosis-associated proteins. β-actin served as a loading control.

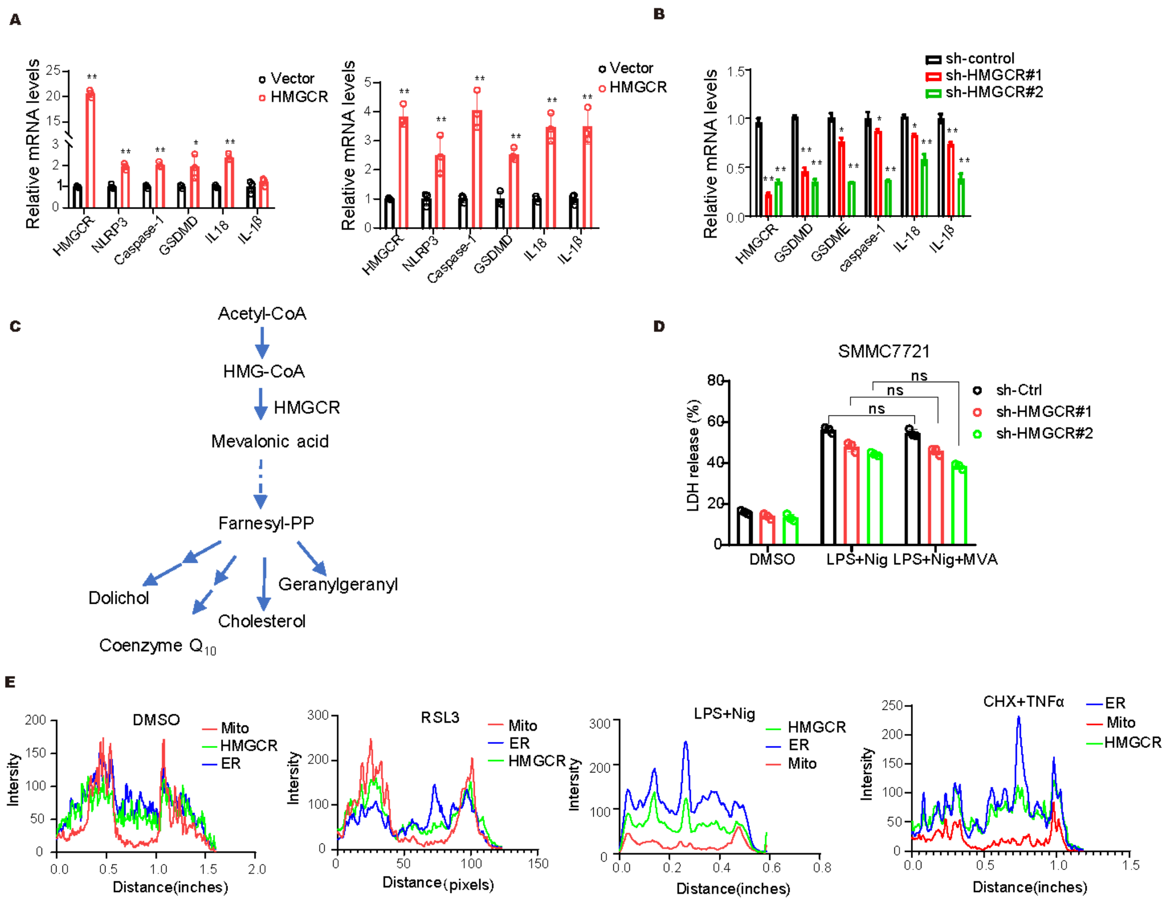

Fig. S2. HMGCR promotes pyroptosis in endoplasmic reticulum and inhibits ferroptosis in mitochondria. (A) RT-RCR assay was used to measure the mRNA expression of the pyroptosis-associated gene in HMGCR overexpression liver cancer. (B) RT-RCR assay was used to measure the mRNA expression of the pyroptosis-associated gene in HMGCR knockdown liver cancer. (C) Schematics of HMGCR mediated mevalonate pathway. (D) SMMC7721 cells with stable HMGCR knockdown were treated with MVA and LPS and Nigericin. The LDH is release by using LDH detection kits. (E) The fluorescence intensity of HMGCR in the mitochondria and endoplasmic reticulum.

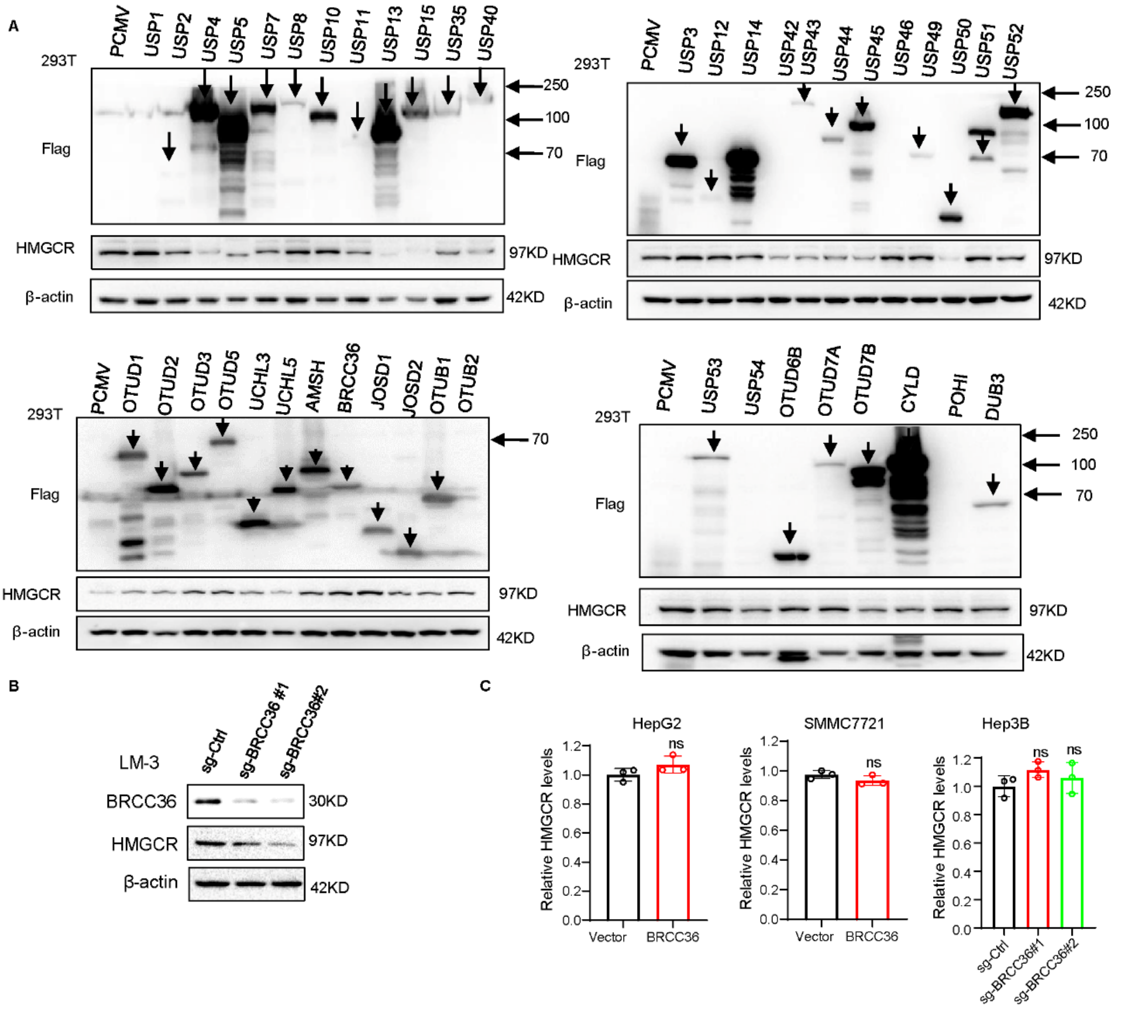

Fig. S3. BRCC36 interacts with HMGCR. (A) 293T cells were transfected with various DUB plasmids, and western blot assay was performed to detect the expression level of Flag and HMGCR. (B) Western blot assay was utilized to detect the expression level of BRCC36 and HMGCR in BRCC36 depleted liver cancer cells. (C) The expression of HMGCR mRNA was detected in HepG2 cells overexpressed or depleted BRCC36 by using RT-RCR assay.

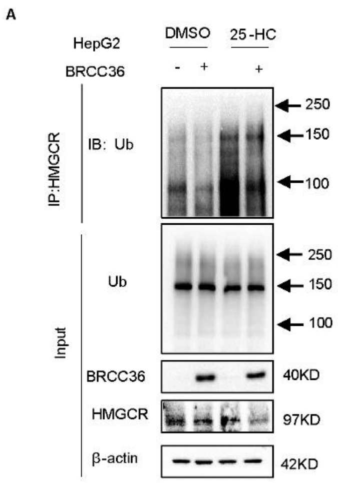

Fig. S4. BRCC36 stabilizes the HMGCR protein through deubiquitination. (A) HepG2 cells overexpressed BRCC36 pretreated 25-HC for 12 h and incubated with MG132 for 12 h. The endogenous HMGCR was immunoprecipitated using an anti-HMGCR antibody and the ubiquitinated HMGCR was detected by an anti-ubiquitin antibody.

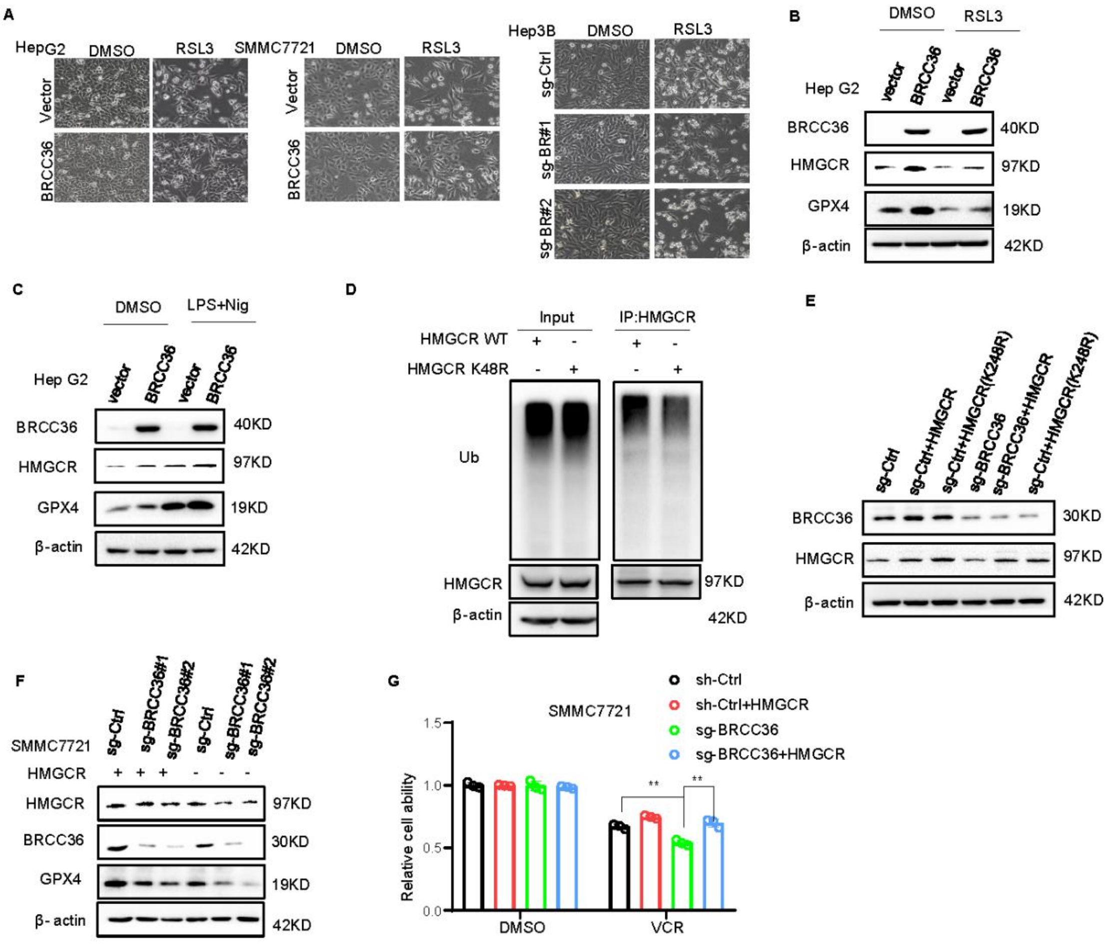

Fig. S5. BRCC36 inhibits ferroptosis and promotes pyroptosis. (A) Representative phase-contrast images of BRCC36-overexpressed or depleted liver cancer cells treated with DMSO or RSL3. Scale bar: 1mm. HepG2 cells overexpressed BRCC36 were treated with RSL3 (B) or LPS and nigericin (C). Western blot assay was performed to detect the expression levels of ferroptosis- and pyroptosis-associated proteins. (D) 293T cells were transfected plasmids of HMGCR or HMGCR (K248R) for 48h, and incubated with MG132 for 12 h. The endogenous HMGCR was immunoprecipitated using an anti-HMGCR antibody and the ubiquitinated HMGCR was detected by an anti-ubiquitin antibody. (E) The BRCC36-knockout cancer cells SMMC7721 were transfected the HMGCR or HMGCR (K248R), and western blot assay was performed to detect the expression of HMGCR and BRCC36. (F) Western blot assay was performed to detect the expression levels of BRCC36 and HMGCR in SMMC7721 cells. β-actin served as a loading control. (G) The cell viability of BRCC36-depleted SMMC7721 cells, with or without HMGCR re-expression, were measured after apoptosis trigger vincristine (VCR).

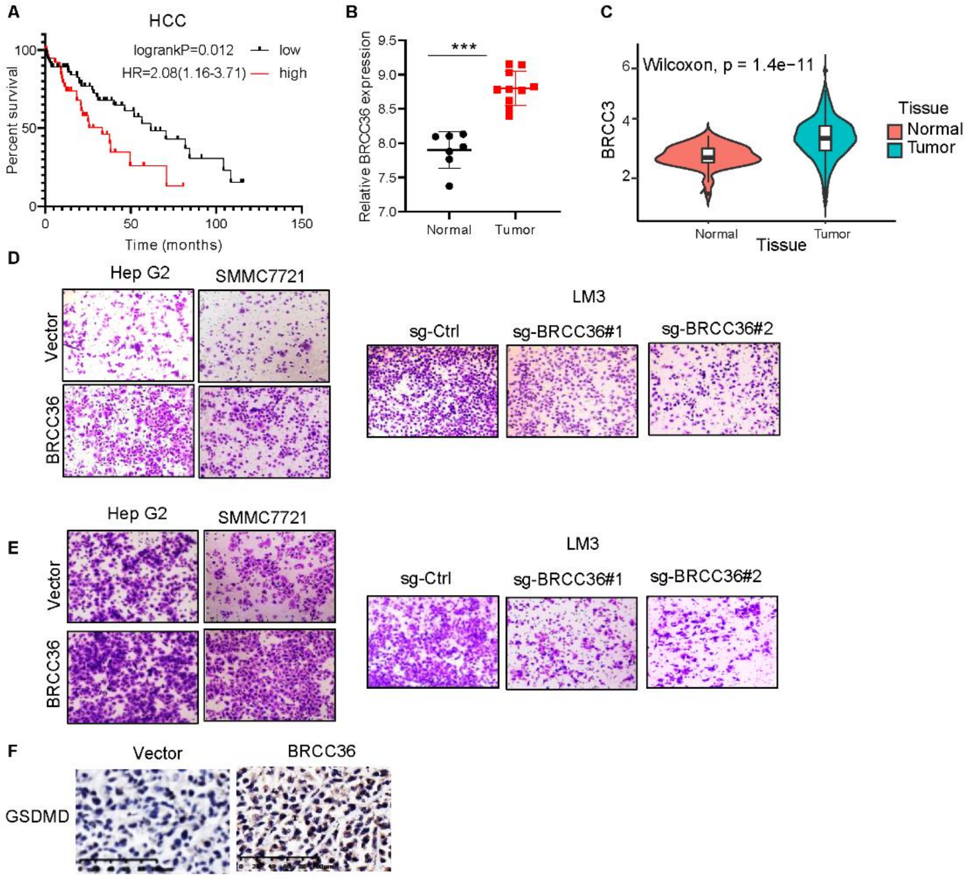

Fig. S6. BRCC36 functions as an oncogene in hepatic carcinoma. (A) Kaplan–Meier curves analyzed the survival rate associated with BRCC36 expression in HCC sample. (B) The expression of BRCC36 in peripheral blood mononuclear cells of hepatocellular carcinoma by using GEO datasets analysis. (C) TCGA analysis of BRCC36 mRNA expression in liver cancer samples and paracancerous normal tissues. (D) The image of transwell migration in HepG2, SMMC7721, and LM3 cells stably overexpressing BRCC36 or depleting BRCC36. (E) The image of transwell invasion in HepG2, SMMC7721, and LM3 cells stably overexpressing BRCC36 or depleting BRCC36. (F) The images of immunohistochemical staining to assess the cell proliferation and the expression level of GSDMD in tumor samples.

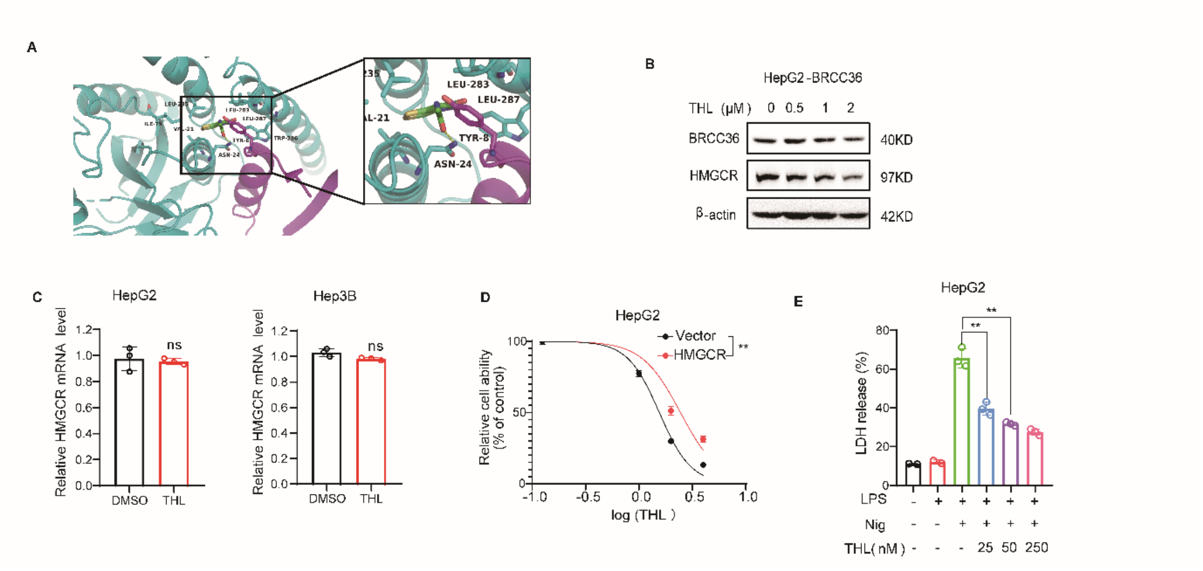

Fig. S7. Thiolutin weakens the interaction between BRCC36 and HMGCR and inhibits tumor growth in HCC. (A) Thiolutin can block interaction between BRCC36 and Abraxas2. The docked pose of thiolutin binding to BRCC36, with its formyl moiety forming hydrogen bonding to BRCC36 Asn 24, sterically clash with Abraxas2 TYR 8. BRCC36 and Abraxsa2 are shown as cartoon structure colored by cyan and magenta respectively. Thiolutin is shown as stick structure. (B) HepG2 cells overexpressed BRCC36 were treated with THL and western blot assay was performed to detect the expression levels of BRCC36 and HMGCR proteins. β-actin served as a loading control. (C) RT-qPCR was used to detect HMGCR mRNA levels in Hep3B and HepG2 cells treated with THL or DMSO. (D) The HepG2 cells were treated with THL after overexpressing HMGCR and the cell viability was detected. (E) HepG2 cells pretreated with THL were stimulation with LPS and Nigericin and measured the LDH release by using LDH detection kits.
